# Label-Free Proteomics Reveals the Molecular Mechanism of Subculture Induced Strain Degeneration and Discovery of Indicative Index for Degeneration in *Pleurotus ostreatus*

**DOI:** 10.3390/molecules25214920

**Published:** 2020-10-24

**Authors:** Weiwei Zhu, Jinbo Hu, Jingliang Chi, Yang Li, Bing Yang, Wenli Hu, Fei Chen, Chong Xu, Linshan Chai, Yongming Bao

**Affiliations:** 1 School of Bioengineering, Dalian University of Technology, Dalian 116024, China; zhuweiwei@vip.163.com; 2 Microbial Research Institute of Liaoning Province, Chaoyang 122000, China; chijingliang@sina.com (J.C.); liyang_0223@163.com (Y.L.); chenfei3033@vip.sina.com (F.C.); sss646@163.com (C.X.); lschai2019@163.com (L.C.); 3 Laboratory of Photosynthesis and Environment, CAS Center for Excellence in Molecular Plant Sciences, Chinese Academy of Sciences, Shanghai 200032, China; hujinbo@sibs.ac.cn (J.H.); yangbing@sibs.ac.cn (B.Y.); 4 University of Chinese Academy of Sciences, Beijing 100049, China; 5 Core Facility Center, CAS Center for Excellence in Molecular Plant Sciences, Chinese Academy of Sciences, Shanghai 200032, China; wlhu@cemps.ac.cn; 6 School of Ocean Science and Technology, Dalian University of Technology, Panjin 124021, China

**Keywords:** *pleurotus ostreatus*, proteomics, subculture, strain degeneration, DNA damage repair, homologous recombination

## Abstract

*Pleurotus ostreatus* is one of the widely cultivated edible fungi across the world. Mycelial subculture is an indispensable part in the process of cultivation and production for all kinds of edible fungi. However, successive subcultures usually lead to strain degeneration. The degenerated strains usually have a decrease in stress resistance, yield, and an alteration in fruiting time, which will subsequently result in tremendous economic loss. Through proteomic analysis, we identified the differentially expressed proteins (DEPs) in the mycelium of *Pleurotus ostreatus* from different subcultured generations. We found that the DNA damage repair system, especially the double-strand breaks (DSBs), repairs via homologous recombination, was impaired in the subcultured mycelium, and gradual accumulation of the DSBs would lead to the strain degeneration after successive subculture. The TUNEL assay further confirmed our finding about the DNA breaks in the subcultured mycelium. Interestingly, the enzyme activity of laccase, carboxylic ester hydrolase, α-galactosidase, and catalase directly related to passage number could be used as the characteristic index for strain degeneration determination. Our results not only reveal for the first time at the molecular level that genomic instability is the cause of degeneration, but also provide an applicable approach for monitoring strain degeneration in process of edible fungi cultivation and production.

## 1. Introduction

*Pleurotus ostreatus* (*P. ostreatus*) is a kind of worldwide edible fungus that has been cultivated for centuries [1,2]. In agricultural production, mycelial subculture is the widely adopted method for the preservation and reproduction of the strain [3]. However, strain degeneration is inevitable after successive subcultures [4]. In the vegetative stage, the degenerated strain shows not only decreased and nonsynchronous mycelial growth rate, but also excessive pigment accumulation, resembling the stress-related phenotype [3,4,5]. In the reproductive stage, the degenerated strain shows an increased pigment production, declined number of primordium and distorted fruiting body, or even no fruiting body [6,7,8,9], which will lead to great economic loss. Strain degeneration is not a sudden occurrence, but a gradual trans-generational accumulation from the individual to the population through successive subcultures or infected by a virus [10,11]. Unfortunately, in the process of agricultural production, the degenerated mycelia were morphologically indistinguishable from the normal ones, and the phenotype of degeneration will not be apparent until the final stage of mushroom cultivation, when the economic losses cannot be prevented. Until today, the molecular mechanism of the strain degeneration is still unknown [12], which makes it more difficult to detect the degeneration of mycelium before agricultural production. Therefore, an understanding of the molecular mechanism of strain degeneration is urgently needed for directing an effective and reliable method to detect and distinguish the degenerated strains in the early stage of agricultural production. More importantly, elucidating the molecular mechanism of edible fungi degeneration is of great significance for better understanding the developmental diversification and evolution of the fungal kingdom.

To explore the underlying molecular mechanism of strain degeneration, we chose the subcultured mycelium of the model fungi *P. ostreatus*, from the 1st (named P1) and 10th (named P10) generation to elucidate the differentially expressed proteins (DEPs) through label-free proteomics. DEPs (501) with 277 up-regulated and 224 down-regulated in P10 were obtained, compared with P1. GO (Gene Ontology) annotation revealed that the DEPs were mainly involved in the metabolic process and cellular process through the molecular function of catalytic and binding. Kyoto Encyclopedia of Genes and Genomes (KEGG) analysis revealed that most DEPs were related to the process of metabolism, genetic information processing, environmental information processing, and cellular processes. Further enrichment analysis of the up-regulated and down-regulated DEPs provided more information about the biological processes and pathways involved in the strain degeneration. We found that the excision repair pathway was activated, while the double-strand break repair via homologous recombination and the cell cycle were impaired in P10, suggesting that P10 accumulated more DNA damages and the cell had to impose a delay of the cell cycle in response to the genomic damages. To validate the accuracy of our proteomic result, the enzyme activity of several enzymes was performed in more generations and the results were in complete agreement with the proteomic results. Furthermore, we conducted the TUNEL assay to further confirm the existence and accumulation of the DNA damages in the subcultured mycelium. Most importantly, an applicable approach was set up based on our proteomic results for monitoring the strain degeneration in the process of edible fungi cultivation and production.

## 2. Results

### 2.1. High-Throughput Proteomics Identification and Quantitative Analysis of the Hyphae from P1 and P10 in P. ostreatus

**Figure 1:**
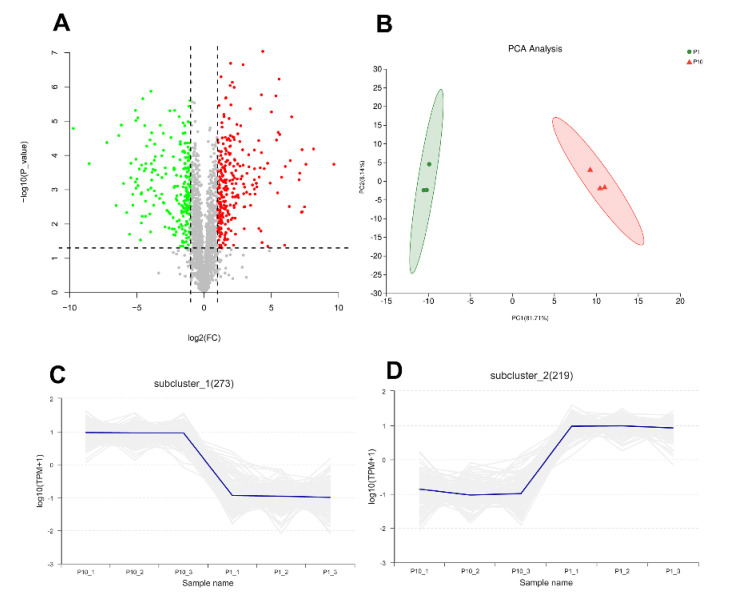
Overview of the proteomic result. The volcano plot (**A**) showed 277 up-regulated differentially expressed proteins (DEPs) and 224 down-regulated DEPs. Principal component analysis (PCA) (**B**) showed good repeatability of each biological replicate. Consistent expression trend of 273 proteins in up-regulated DEPs (**C**), and 219 proteins in down-regulated DEPs (**D**).

In order to explore the molecular mechanism of degeneration of subcultured mycelium of *P. ostreatus*, mass spectrometry-based label-free quantitative proteomics was performed to analyze the differentially expressed proteins (DEPs) from the mycelium of P1 and P10. In the results, the peptide length distribution and protein sequence coverage distribution showed a good instrumental condition and experimental procedure (Figure S1A,B). A total of 2062 proteins from 13,569 peptides and 68,983 PSMs (peptide spectrum match) with quantitative information were identified in our experiment under the criteria of both peptide and protein FDR < 0.01. Compared with P1, there are 277 up-regulated proteins and 224 down-regulated proteins in P10 (Fold Change > 2 or <0.5 and *p-*value < 0.05), respectively (Figure 1A, Figure S1C). Principal component analysis (PCA) and the correlation between samples indicated good repeatability of each biological replicate in the same group (Figure 1B, Figure S1D). The expression pattern of 492 proteins of the 501 DEPs showed consistent expression levels in P1 and P10, respectively (Figure 1C,D). All the identified proteins including the detailed information, the up-regulated, and down-regulated proteins are shown in Table S1.

### 2.2. GO and KEGG Annotation of DEPs in P. ostreatus

**Figure 2:**
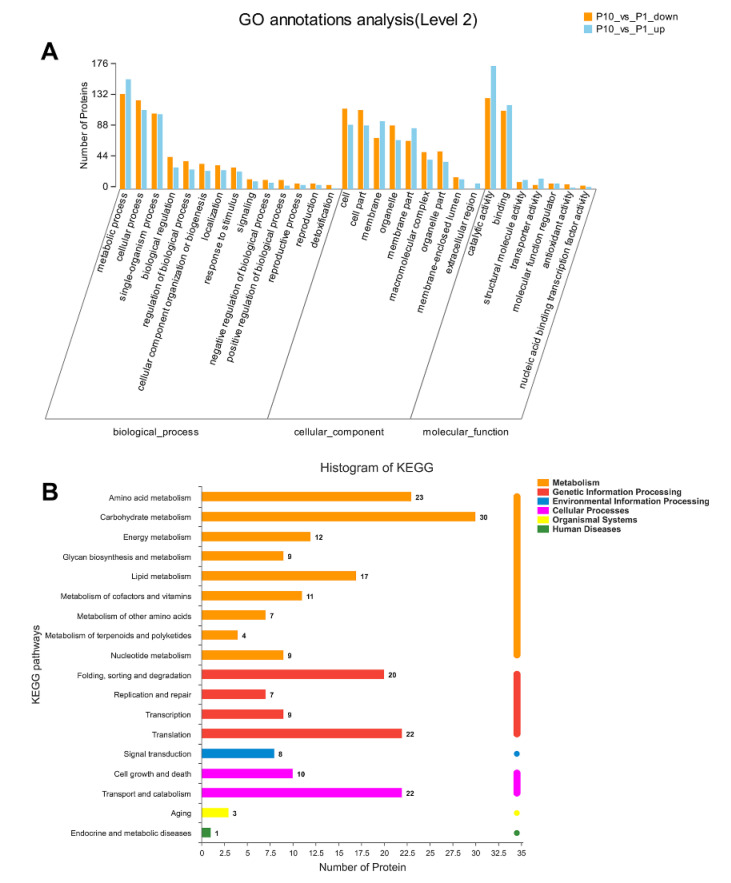
GO and Kyoto Encyclopedia of Genes and Genomes (KEGG) annotation of all the DEPs in *Pleurotus ostreatus*. GO annotation of the DEPs in level 2 with the up-regulated proteins in orange column and down-regulated proteins in blue column, respectively (**A**). KEGG pathway of all the DEPs was mapped into different processes (**B**).

GO and KEGG annotation are basic bioinformatic annotation for the function of proteins, which can provide a general overview of the DEPs, especially for the non-model organisms in three aspects of biological process (BP), molecular function (MF), and cellular component (CC). In our analysis, all the DEPs were classified into different level 2 terms of BP, MF, and CC through GO annotation and the top 30 terms were shown in Figure 2A. In the BP category, terms of metabolic process, cellular process, and single-organism process contain the most abundant DEPs. In the CC category, the cell, cell part, and membrane were the top three terms according to the involving number of DEPs. In the MF category, the terms of catalytic activity, binding, and structural molecule activity showed dominant distribution in the DEPs. The GO annotation result indicated that the DEPs in our experiment were mainly involved in the metabolic process and cellular process through the molecular function of catalytic and binding.

The Kyoto Encyclopedia of Genes and Genomes (KEGG) database was applied to annotate the involving pathways of the DEPs in our experiment. As a result, we found that most DEPs were related to the process of metabolism, genetic information processing, environmental information processing, and cellular processes (Figure 2B). Among these processes, the pathways of carbohydrate metabolism, amino acid metabolism, translation, transport and catabolism, and folding ranked in the top five DEPs involving pathways, which is consistent with the biological process (BP) of the GO annotation.

### 2.3. GO Enrichment Analysis Revealed an Asynchronous Repair System in P10 in Response to DNA Damages

**Figure 3:**
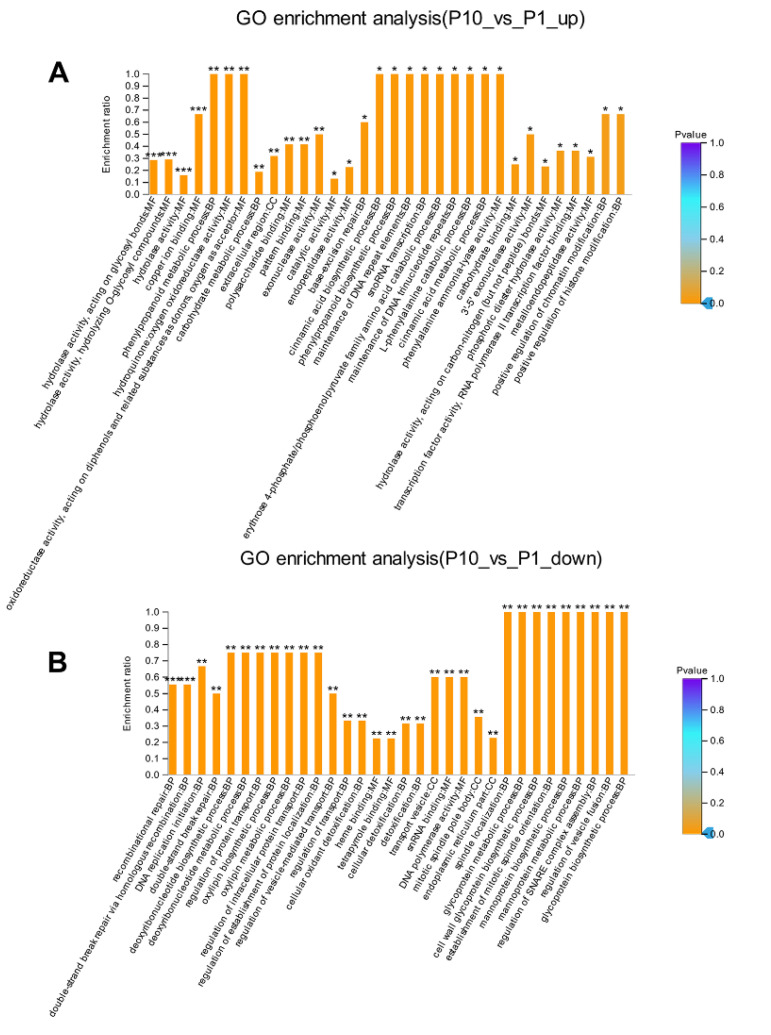
GO enrichment analysis of the up-regulated (**A**) and down-regulated (**B**) DEPs. * *p*-value < 0.05, ** *p*-value < 0.01, *** *p*-value < 0.001.

The enrichment analysis can explore the implied molecular mechanism of the DEPs compared with the background information, which can provide a direct clue for the underlying mechanism of the strain degeneration caused by successive subcultures. Through GO enrichment analysis, several important terms belonging to the molecular function were highly enriched in up-regulated DEPs (Figure 3A, Table S2), including hydrolase activity, acting on glycosyl bonds (*p* < 0.001); hydrolase activity, hydrolyzing O-glycosyl compounds (*p* < 0.001); hydrolase activity (*p* < 0.001); copper ion binding (*p* < 0.001). Most DEPs with the function of hydrolase activity located in the up-regulated terms indicated that P10 may have high catabolic metabolism compared with P1, which was also consistent with previous observations in other filamentous fungi [13,14]. While in the down-regulated DEPs, significantly enriched terms were concentrated in the biological process (Figure 3B, Table S3), including the recombinational repair (*p* < 0.001), double-strand break repair via homologous recombination, DNA replication initiation (*p* < 0.01), double-strand break repair (*p* < 0.01), deoxyribonucleotide biosynthetic, and metabolic process (*p* < 0.01). These terms were all related to the process of DNA replication and damage repair, suggesting that DNA replication was delayed, probably due to the down-regulated DNA damage repair system, especially the recombination repair in P10.

#### 2.3.1. Base Excision Repair System Was Activated in the Subcultured Mycelium

There are many kinds of endogenous DNA damages, either from the spontaneous mutation or introduced by the process of DNA replication [15,16]. Accordingly, the organisms have evolved different kinds of repair systems in response to these damages [17]. Excision repair is the most common and effective method that the cell would adopt to repair the helix distortion and non-helix distortion damages by nucleotide excision repair (NER) and base excision repair (BER), respectively [18,19,20]. Many damages can be repaired by excision repair except for the double-strand break. In our analysis, the base-excision repair (*p* < 0.05) in the BP was highly enriched in the up-regulated DEPs (Figure 3A), indicating that mutations in the mycelium accumulated through successive subcultures could be (or at least partially) repaired by the BER system through either short-patch pathway or long-patch pathway (or both) [21,22]. This point was consistent with the GO enrichment term that the hydrolase activity acting on the glycosyl bonds was significantly enriched (*p* < 0.001) in the up-regulated DEPs (Figure 3A). The damaged DNA bases would be excised firstly by the catalyzation of N-glycosidases, which act directly on the glycosyl bonds through the hydrolase activity [23,24]. Then the endonuclease APE1 homolog (homologs) would introduce an incision on the sugar-phosphate backbone near the damaged bases [25,26,27]. The next excision repair steps were either through the short-patch pathway under the catalyzation of DNA polymerase β or through the long-patch pathway under the catalyzation of DNA polymerase δ/ε and other assistant components [28,29]. In our study, the enriched exonuclease activity (*p* < 0.01), the N-Glycan biosynthesis (*p* < 0.05), and various types of N-glycan biosynthesis (*p* < 0.05) in the up-regulated GO and KEGG results further confirmed the involvement of the long-patch pathway in the mycelial DNA damages repair process (Figure 3A). Because the long-patch pathway would remove 2–10 nucleotides on both sides of the damaged bases and resynthesize each nucleotide unit according to the complementary strand under the catalyzation of DNA polymerase δ/ε, and the DNA polymerase δ/ε naturally has the exonuclease activity [30,31,32].

#### 2.3.2. Double-Strand Break (DSB) Repair System Impaired in the Subcultured Mycelium

DNA double-strand break (DSB) is another type of DNA damage. The sugar-phosphate backbone of both strands would break at the same or very close point [33]. DNA double-strand break (DSB) is the most deleterious lesion arising in the genome of all eukaryotic organisms, which can severely affect the genomic integrity and stability [34]. It occurs normally during DNA replication, mitosis, and meiosis. However, DSBs cannot be repaired by normal repair mechanisms, like nucleotide excision repair (NER) and base excision repair (BER) [35,36]. If the double-strand breaks cannot be repaired efficiently, the free broken strands can lead to translocations, even deletions, which will cause many developmental problems, even death [37,38]. Fortunately, the eukaryotic organisms have evolved efficient mechanisms that can sense and quickly respond to DSB events and ultimately repair them. Homologous recombination (HR) and non-homologous end-joining (NHEJ) are two main pathways that the cell would adopt to repair the double-strand breaks damages [39]. Homologous recombination (HR) can repair the double-strand breaks faithfully in the S and G2 phase of the cell cycle, while the non-homologous end-joining (NHEJ) can repair the double-strand breaks throughout the whole cell cycle in an error-prone manner [40,41,42]. In our study, the GO enrichment showed a significant difference in the terms regarding recombinational repair (*p* < 0.001), double-strand break repair via homologous recombination (*p* < 0.001), and double-strand break repair (*p* < 0.01) in the down-regulated DEPs (Figure 3B), indicating that the homologous recombination (HR) repair pathway in response to the DSBs in the subcultured mycelium was impaired. Strain degeneration would happen when more double-strand breaks got accumulated as the generations of subculture increased. It could be the reason why the strain applied in production often shows degeneration phenotype after successive subcultures.

### 2.4. KEGG Enrichment Analysis Revealed the Cell Cycle Was Stalled in P10

**Figure 4:**
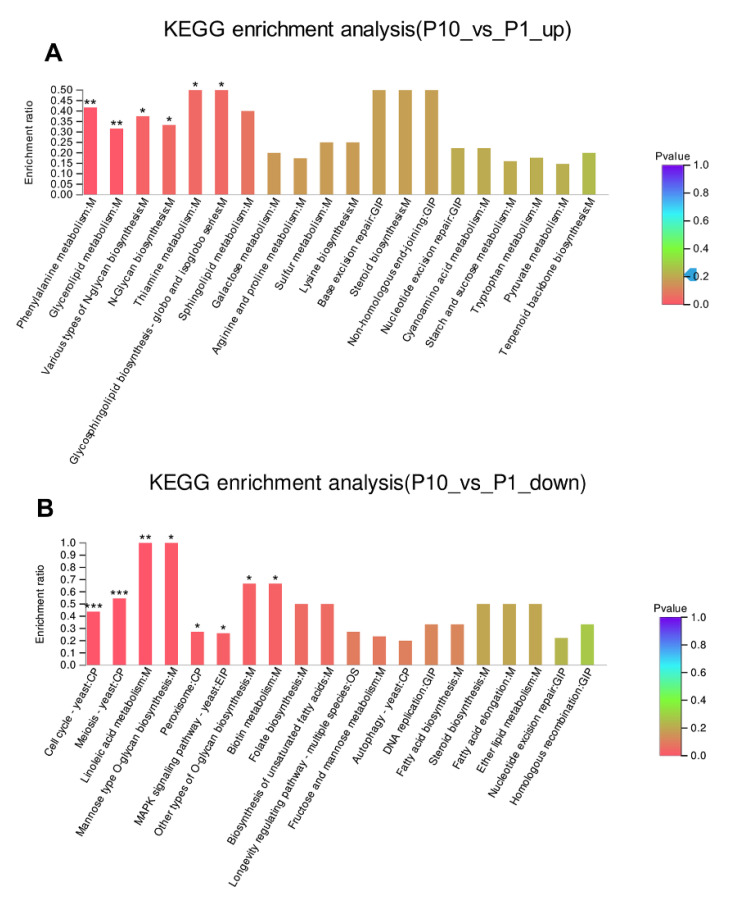
KEGG enrichment of the up-regulated (**A**) and down-regulated (**B**) DEPs. * *p*-value < 0.05, ** *p*-value < 0.01, *** *p*-value < 0.001.

The DEPs were further used as the query proteins and all the identified proteins in our experiment as the background to conduct the KEGG enrichment analysis (Figure 4A, B). It was found that the up-regulated DEPs were enriched in phenylalanine metabolism (*p* < 0.01), glycerolipid metabolism (*p* < 0.01), various types of N-glycan biosynthesis (*p* < 0.05), N-glycan biosynthesis (*p* < 0.05), thiamine metabolism (*p* < 0.05), and glycosphingolipid biosynthesis (*p* < 0.05). In addition, the base excision repair and non-homologous end-joining were also observed in the up-regulated KEGG enrichment (Figure 4A). This result was also consistent with the result of top GO enrichment terms about the up-regulated DEPs in the terms of molecular function and biological process (Figure 3A). Subsequently, we made the KEGG enrichment analysis of the down-regulated DEPs. Compared with the pathways that participated in the up-regulated DEPs, more pathways were found highly enriched in the down-regulated DEPs (Figure 4B). Among them, the most significantly enriched pathways were distributed in cell cycle (*p* < 0.001), meiosis (*p* < 0.001), linoleic acid metabolism (*p* < 0.01), mannose type O-glycan biosynthesis (*p* < 0.05), and peroxisome (*p* < 0.05). In addition, the DNA replication pathway, homologous recombination pathway could also be observed in the down-regulated DEPs (Figure 4B), which was consistent with the GO enrichment of the down-regulated DEPs (Figure 3B). This result indicated that the cell cycle delayed greatly in P10 after successive subcultures.

### 2.5. Enzymatic Activity Assays Showed Consistent Results with the Quantitative Expression of the DEPs 

**Figure 5:**
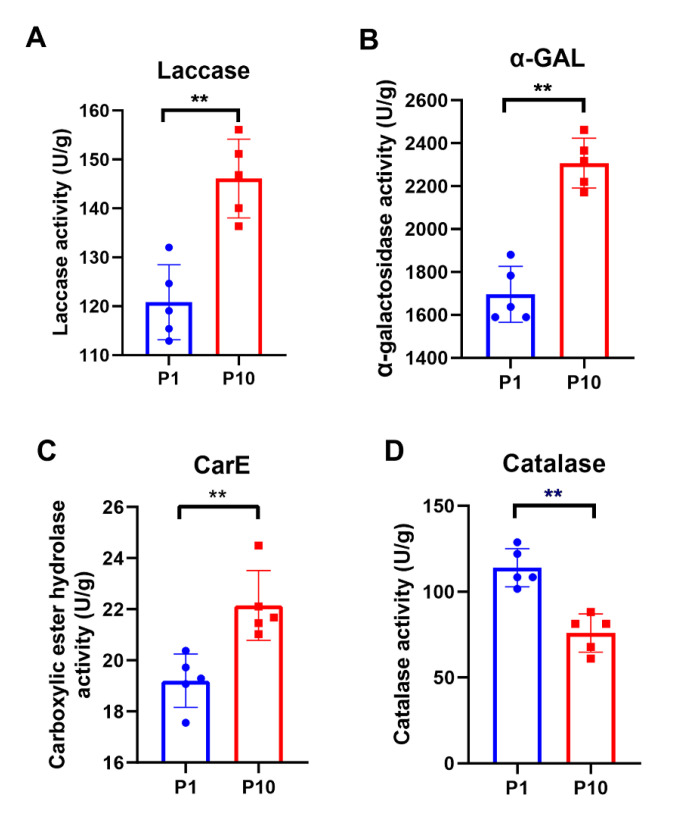
Enzymatic assay of the representative metabolic enzymes in P1 and P10. Each assay included five biological replicate and all the statistical analyses were performed with GraphPad Prism 8 software, using a non-parametric Mann–Whitney test (** *p*-value  ≤  0.0079).

GO and KEGG enrichment results showed that there were many metabolic differences between P1 and P10. Therefore, several representative metabolic enzymes were selected to test their activity in P1 and P10, respectively. Each assay included five biological replicates. As known, laccases are particularly widespread in ligninolytic basidiomycetes, participating in the catalytic processes of lignin degradation. So, the enzymatic activity of the laccase was measured in P1 and P10. The result showed that the laccase activity of P10 was indeed higher than that of P1 (Figure 5A), which was consistent with both our quantitative data (Table 1) and GO enrichment result of the up-regulated DEPs in the term of copper ion binding; hydroquinone: oxygen oxidoreductase activity; oxidoreductase activity, acting on diphenols, and related substances as donors and oxygen as an acceptor (Figure 3A), because the copper ion plays a central role in the function of laccase during the catalytic process of phenolic compound oxidation. The enzymatic activity of α-galactosidase (α-GAL) and carboxylic ester hydrolase (CarE) were then detected, which were also ubiquitous enzymes with hydrolase activity. Consistent with our enrichment analysis and quantitative expression (Table 1), both enzymes showed higher activity in P10 (Figure 5B, C). Catalase is one of the most important enzymes in the peroxisome, but only one catalase was identified in our study, which belonged to the peroxisome pathway in the down-regulated DEPs. After catalase activity assay consistent with the quantitative result was obtained (Table 1), P10 displayed a lower catalase activity than P1 (Figure 5D). Collectively, the accuracy of our proteomic data and bioinformatic analysis was further confirmed by the enzymatic activity assays in P1 and P10.

### 2.6. TUNEL Assay Further Confirmed the Accumulation of DNA Damages from the 1st Generation to the 15th Generation

**Figure 6:**
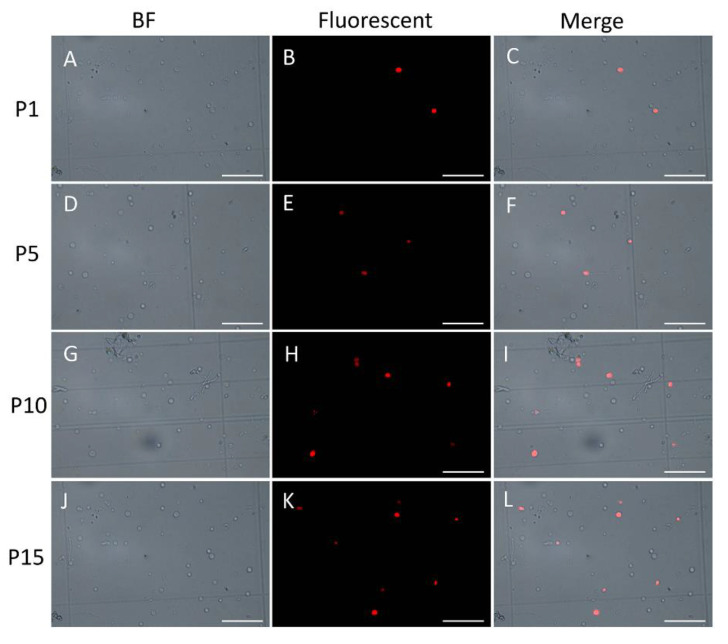
TUNEL assay of the subcultured mycelium from different generations. **A**–**C**, 1st generation; **D**–**F**, 5th generation; **G**–**I**, 10th generation; **J**–**L**, 15th generation. Bars = 10 μM.

**Figure 7:**
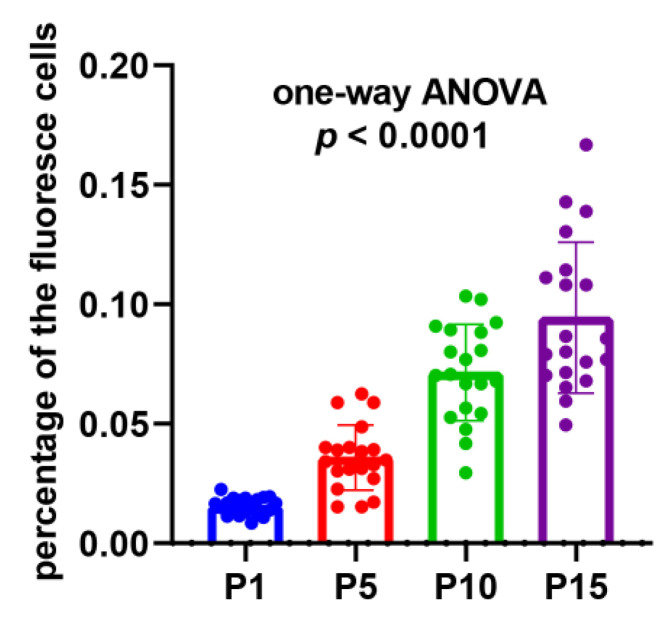
The percentage of the fluorescence cells in all the mycelial cells in different subcultured generations. Twenty replicates were included in each generation and the *p-*value of one-way ANOVA < 0.0001.

In order to demonstrate the existence and persistence of DNA damages from the 1st generation to the 10th, even the 15th generation, we performed the TUNEL assay of the mycelial cells from the 1st, 5th, 10th, and 15th generations after the lywallzyme digestion. The mycelial cells of the 1st generation showed a little fluoresce staining (Figure 6A–C). As the passage number increased, more mycelial cells showed fluoresce staining (Figure 6D–L). We made a statistical analysis of the percentage of the cells with fluoresce staining in all mycelial cells (Figure 7). The average percentage of fluoresce staining mycelial cells at the 1st generation kept at 1.56%, while increased to 3.59% at the 5th generation, 7.14% at the 10th generation, subsequently to 9.44% at the 15th generation (Figure 7). The TUNEL assay demonstrated that the DNA damages were widespread in the subcultured mycelium, and with the increase of subculture generations, DNA damage could be accumulated and persisted, eventually leading to the strain degradation.

### 2.7. Application of the Enzymatic Activity Assay for Strain Degenerative Determination in the Process of P. ostreatus Production

**Figure 8:**
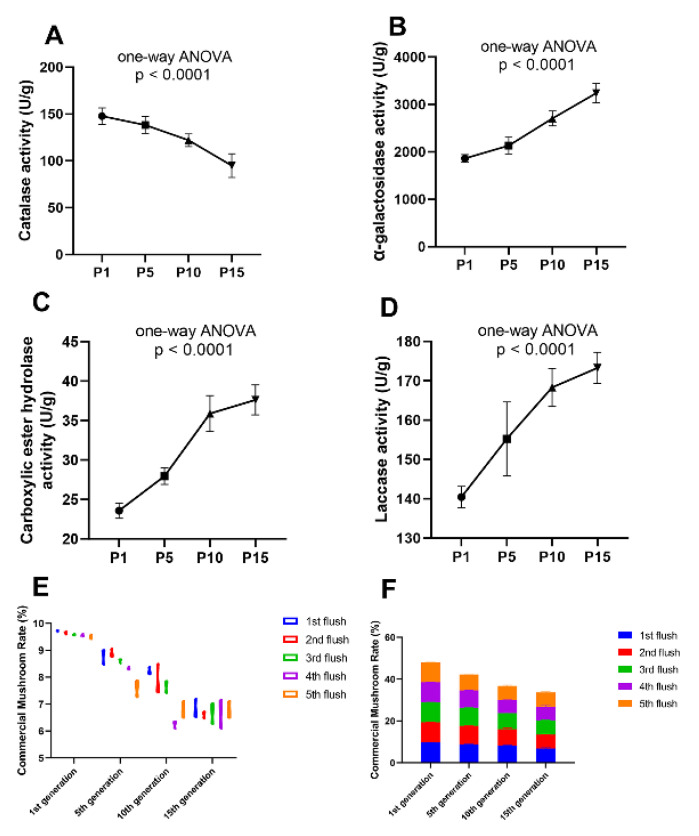
Enzymatic activity assay of the metabolic enzymes in different subcultured generations and the ultimate yield after fruiting. The catalase activity (**A**) decreased with the increase of subcultured generations, the α-galactosidase (**B**), carboxylic ester hydrolase (**C**), and laccase activity (**D**) increased with the increase of subcultured generations. The commercial mushroom rate (CMR) was gradually decreased with the increase of subcultured generation in each (**E**) and all five flushes (**F**).

To prevent the economic loss caused by the strain degeneration, a reliable method to predict the degeneration event of the edible fungi at the stage of hyphae is urgently needed in the process of the edible mushroom. The assay of enzymatic activity is not only convenient to operate but also economical to apply. Therefore, the enzymatic assay method was applied to more subcultured mycelium of *P. ostreatus* from different generations to test the validity and feasibility. The *P. ostreatus* subcultured mycelium from the 1st, 5th, 10th, 15th generations were selected, named P1, P5, P10, and P15, respectively. Enzymatic activity assays of the mycelium from these generations were made ahead of inoculation and each assay included five biological replicates. The ultimate yield of these mycelia from different generations was calculated after fruiting. The enzymatic activity of laccase, carboxylic ester hydrolase, α-galactosidase, and catalase was detected, which were previously proved to have different quantitative expression and enzymatic activity in our proteomic result in P1 and P10. Among the four enzymes, the catalase showed a declined enzymatic activity with the increase of subcultured mycelial generations (Figure 8A), conversely, the other three enzymes showed an enhanced enzymatic activity as increased generations of the subcultured mycelium were tested (Figure 8B–D). Then, the mycelium from different subcultured generations (1st, 5th, 10th, 15th) was inoculated into culture-bags and the yield was calculated, respectively. The CMR (commercial mushroom rate: the dry weight of the commercial mushroom/dry weight of the culture bag) was referenced to describe the yield of subcultured mycelium from different generations. We found that the CMR gradually decreased as the subcultured generation increased in nearly all of the five flushes of mushrooms, respectively (Figure 8E,F). At the same time, the variation between replicates became larger with the increase of subcultured generations (Figure 8E). These results showed a precise quantitative result of the yield in different generations of subcultured mycelium in production, suggesting that the yield of the mushroom would be decreased as the subcultured mycelial generations increased. Specific enzymatic activity in the mycelium had a direct and close relationship with degenerative status of the subcultured mycelium and the subsequent yield formation in a positive or negative manner, indicating that the enzymatic activity could be an effective marker to predict the degenerative condition of the subcultured mycelium ahead of the fruiting stage, which can avoid tremendous economic loss.

## 3. Discussion

Degeneration is a widespread problem in the process of edible fungi cultivation. Our research about the degeneration mechanism is quite relevant to mushroom production. Through the isolation and analysis of DEPs from the 1st and 10th generation of subcultured mycelium of the *P. ostreatus*, it was concluded that the cell cycle of the degenerative mycelium was delayed in response to the impaired DNA damage repair system, especially the double-strand break repair, which usually leads to severe instability of the genome.

### 3.1. Where Are the DNA Damages in the Cells of Hyphae Coming From?

There are many kinds of reasons which can lead to the DNA damage, basically including intrinsic inducers (spontaneous base modifications, replication errors, endogenous oxygen radicals) and exogenous inducers (exogenous chemical mutagens, ultraviolet radiation, ionizing radiation) [43]. The exogenous inducers are usually much less frequent during the life cycle of all organisms except for exposure to some extreme conditions. Most of the DNA damages are from intrinsic ways, which can mainly arise from oxidative damages during metabolism [16]. For example, it has been reported that one single human cell is subject to approximately 70,000 lesions per day [44]. However, no matter the intrinsic or exogenous inducers, they have always behaved as a selecting pressure during the evolutionary process [16,45,46,47]. It has been clear that the cells have acquired mechanisms and enzymes during long-time evolution that can repair the DNA damages and thereby sustain the genomic stability [17,44]. In the natural condition, the fruiting fungi usually finishes the life cycle in a short period through several steps: germination of the spores, primary mycelium, secondary mycelium, hyphal knot, primordium, and mature fruiting body [48]. During those processes, the hyphae of the secondary mycelium can be manually preserved and cultured separately, providing the infinitely amplified “seeds” for mushroom production [4,9,49]. Therefore, subculture could be used to reproduce the hyphae from secondary mycelium to obtain enough “seeds” for more inoculation in the process of production. In fact, subculture is an indispensable process for hyphae amplification and asexual reproduction. Each time of amplification represents a replication of the whole diploid genome of the mycelium through mitosis. DNA damages gradually accumulate during the process of genomic replication. The more times of DNA replication, the more damages accumulate, the more degenerative the fungi become. It is the reason why natural growing edible fungi do not show the degenerative phenotype, because the secondary hyphae of mycelium do not experience the process of subculture.

### 3.2. Multiple Level and Coordinated Regulation of the Cellular Processes in Response to DNA Damage through Cell Cycle Regulation, MAPK Signaling, Chromatin Remodeling, and Epigenetic Modification

In normal cells, the DNA DSBs can be swiftly recognized by some sensors and transducers that allow the promotion of hierarchical signaling cascades to transduce the damage signals to many downstream effectors to coordinate the cellular processes including cell cycle, anabolism, and catabolism [50,51]. Early signaling effectors in the DSBs were mainly involved in post-translational modifications through phosphorylation, acetylation, and methylation of the substrates [52,53,54,55,56,57,58]. In our study, the MAPK signaling pathway (*p* < 0.05) in the KEGG analysis was also enriched in the down-regulated DEPs (Figure 4B), suggesting that some of the early effectors that sensed and transduced the DSBs’ signaling were blocked in the subcultured mycelium, which was quite consistent with previous observation in *Podospora anserina* [59]. Those signaling effectors played critical roles in DNA damage checkpoint when the repair system can find and subsequently repair the DNA damages. They can impose a delay in the cell cycle so that the cells harboring DNA damages would obtain enough time to repair these damages that accumulated in the genome [60,61,62,63,64]. In our study, the GO terms and KEGG pathways about DNA replication initiation (*p* < 0.01), deoxyribonucleotide biosynthetic process (*p* < 0.01), deoxyribonucleotide metabolic process (*p* < 0.01), mitotic spindle pole body (*p* < 0.01), spindle localization (*p* < 0.01), establishment of mitotic spindle orientation (*p* < 0.01), cell wall glycoprotein biosynthetic process (*p* < 0.01), cell cycle (*p* < 0.001), and meiosis (*p* < 0.001) were highly enriched in the down-regulated DEPs (Figure 3B & Figure 4B), suggesting that the mycelial cells can sense the DSBs’ damages such that they make a stalling of the cell cycle, but the DSBs cannot be efficiently repaired by the repairing system, especially the homologous recombination pathway (Figure 3B). 

As known, chromatin modifications play a substantial role, not only in transcriptional activation and repression, but also in the process of many kinds of DNA damage repair through regulating the chromatin dynamics [65,66,67,68,69,70]. Epigenetic modifiers including chromatin remodelers, histone modifiers, DNA (de-)methylation enzymes, and even noncoding RNAs regulate the DNA damage response and repair system by affecting chromatin assembly and disassembly, releasing a conformational and biochemical environment from the packaged chromatin for the repair enzymes [71,72,73]. In our study, the GO terms of positive regulation of chromatin modification (*p* < 0.05) and positive regulation of histone modification (*p* < 0.05) were enriched in the up-regulated DEPs, suggesting that the epigenetic modifications in P10 play an active role in regulating the DNA damage repair through chromatin dynamics, which was consistent with not only our result that the base excision repair system was activated in P10 [74,75], but also the previous observation in *Cordyceps militaris* [76]. 

**Figure 9:**
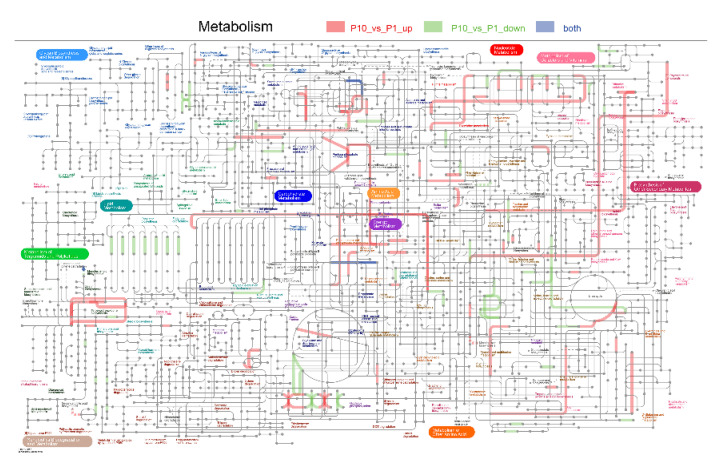
Integrated pathways of the metabolic processes in the DEPs. Pathways labelled with red lines indicated the up-regulated DEPs, green lines indicated down-regulated DEPs, and gray lines indicated that both the up- and down-regulated DEPs were included in it.

Taken together, our results suggested that accurate repair of the DNA damages must depend on precise and sophisticated regulation that orchestrates not only various but also multi-dimensional metabolic processes to coordinate signals from a full set of sensors and transducers in different cellular processes (Figure 9).

### 3.3. Effective Ways of Preventing the Strain Degeneration During Mushroom Production

The hyphae growth of the secondary mycelium is the result of the clamp connection and division of a single hypha cell. Therefore, strictly limiting the length of the subcultured hyphae from secondary mycelium (“seeds”) in the reproductive process during mushroom production equals inhibiting the cell division times of a hypha cell, which can avoid the accumulation of DNA damages caused by DNA replication during the cell division. This method can effectively inhibit the degeneration during the stage of fruiting and in fact, we had applied it in production for several years.

## 4. Materials and Methods 

### 4.1. Mycelium Subculture and Fruiting Body Culture Conditions

*P. ostreatus* strain was obtained from Liaoning Center of Culture Collection (LCCC) and the strain number is LCCC 50563. The mycelium was subcultured on the PDA medium in the tube at 25 ± 1 °C and 55–65% humidity in dark condition. The fruiting body was cultured on medium-containing polyethylene bags as previously described [2].

### 4.2. Protein Extraction, Digestion, and Quantification

About 0.1 g mycelium was collected from the medium and grounded into powder in liquid nitrogen. The pre-cooled acetone (containing 10% trichloroacetic acid) was added into the powder and precipitated overnight at −20 °C. Then centrifuge the mixture at 15,000× *g* for 30 min at 4 °C. The supernatant was discarded and the precipitate was washed with pre-cooled acetone 2 times and centrifuged at 15,000× *g* for 30 min at 4 °C after the second washing. The precipitate was dried at room temperature and grounded into power. About 30 times the volume of SDT buffer (4% SDS, 100 mM Tris-HCl, 1 mM DTT, pH 7.6) was added to the powder, mixed, and boiled for 5 min. The mixture was sonicated shortly and then boiled for another 5 min. After being centrifuged at 14,000× *g* for 30 min, the supernatant was filtered with 0.22 µm filters. The filtrate was quantified with the BCA Protein Quantification Kit (Yeasen, Shanghai, China). 

About 200 μg of protein for each sample was used to conduct the FASP digestion [77]. The peptide content was estimated by UV light spectral density at 280 nm using an extinction coefficient of 1.1 of 0.1% (g/l) solution that was calculated based on the frequency of tryptophan and tyrosine in vertebrate proteins.

### 4.3. LC-MS/MS Analysis

About 1 µg peptide from each replicate was injected for nano LC–MS/MS analysis. The peptide mixture was loaded onto a reverse-phase trap column (Thermo Scientific Acclaim PepMap100, 100 μm × 2 cm, nanoViper C18, Waltham, MA, USA) connected to a C18 reversed-phase analytical column (Thermo Scientific Easy Column, 10 cm long, 75 um inner diameter, 3μm resin) in buffer A (0.1% formic acid) and separated with a linear gradient of buffer B (84% acetonitrile and 0.1% formic acid), at a flow rate of 300 nl/min controlled by IntelliFlow technology. The total time for each sample was 120 min. Mass spectrometry analysis was performed on a Q Exactive mass spectrometer (Thermo Scientific) that was coupled to Easy nLC (Proxeon Biosystems, now Thermo Fisher Scientific) for 120 min. The mass spectrometer was operated in positive ion mode. MS data was acquired using a data-dependent top20 method dynamically choosing the most abundant precursor ions from the survey scan (350–1800 *m*/*z*) for HCD fragmentation. Automatic gain control (AGC) target was set to 3e6, and maximum inject time to 50 ms. Dynamic exclusion duration was 30.0 s. The instrument was run with peptide recognition mode enabled.

### 4.4. Data Processing and Analysis

The MS data was analyzed using the Proteome Discover software version 2.4 (Thermo Scientific) against the uniport database by the filtered organism of *Pleurotus ostreatus PC15* (12,186 sequences, downloaded on 23 October 2018). The following parameters were set. Cys alkylation: Iodoacetamide. Dynamic Modification: Oxidation (M), Acetyl (Protein N-Terminus); Static Modification: Carbamidomethyl (C); Enzyme Name: Trypsin (Full); Max. Missed Cleavage Sites: 2; Precursor Mass Tolerance: 10 ppm. Build summary of all the quantitative proteins and peptides was established by both protein and peptide FDR < 0.05.

### 4.5. Bioinformatic Analysis

All the bioinformatic analysis was conducted by the One-Step Majorbio Cloud Bioinformatic Analysis Platform (https://cloud.majorbio.com).

### 4.6. Enzymatic Activity Assay

The enzymatic activity assays were made by using the Enzymatic Assay Kit (Solarbio, Beijing, China) and following the instructions. The catalog number for each enzymatic assay was Laccase (BC1630); CarE (BC0840); α-GAL (BC2570); Catalase (BC0200).

### 4.7. TUNEL Assay

The mycelium from different subcultured generations were digested by lywallzyme for 5 h to remove the cell wall and then the steps were followed of One Step TUNEL Apoptosis Assay Kit (Beyotime, C1089).

## 5. Conclusions

Our results reveal that the homologous recombination repair system is blocked in the subcultured hyphae of *P. ostreatus* and the inefficient repair of the double-strand breaks can lead to the degeneration of the strain. A reliable method we developed could be applied in the strain degeneration determination through the assay of enzymatic activity of hyphae in front of the fruiting stage in the process of cultivation and production, which can be a reference for more degenerative determination of the edible fungi.

## Figures and Tables

**Table 1:** Quantitative expression of the metabolic enzymes in the DEPs.

Accession	Description	P10/P1	*p* Value
A0A067NEP6	Laccase OS = *Pleurotus ostreatus* PC15 OX = 1137138 GN = LACC10 PE = 3 SV = 1	40.59462	1.83 × 10^−6^
A0A067NLM3	Laccase OS = *Pleurotus ostreatus* PC15 OX = 1137138 GN = LACC2 PE = 3 SV = 1	19.24952	1.69 × 10^−6^
A0A067NMI4	Small subunit of laccase POXA3a OS = *Pleurotus ostreatus* PC15 OX = 1137138 GN = PLEOSDRAFT_1067572 PE = 4 SV = 1	13.86408	0.001334
A0A067NQH1	Laccase OS = *Pleurotus ostreatus* PC15 OX = 1137138 GN = LACC6 PE = 3 SV = 1	2.9906	2.97 × 10^−5^
A0A067NW26	Alpha-galactosidase (Fragment) OS = *Pleurotus ostreatus* PC15 OX = 1137138 GN = PLEOSDRAFT_1035175 PE = 3 SV = 1	6.665047	0.000484
A0A067NTX6	Alpha-galactosidase OS = *Pleurotus ostreatus* PC15 OX = 1137138 GN = PLEOSDRAFT_51341 PE = 3 SV = 1	5.675188	0.000174
A0A067NVN8	Alpha-galactosidase OS = *Pleurotus ostreatus* PC15 OX = 1137138 GN = PLEOSDRAFT_1111105 PE = 3 SV = 1	1.706567	0.025545
A0A067NN26	Carboxylic ester hydrolase OS = *Pleurotus ostreatus* PC15 OX = 1137138 GN = PLEOSDRAFT_170071 PE = 3 SV = 1	13.35758	6.89 × 10^−5^
A0A067NDF5	Carboxylic ester hydrolase OS = *Pleurotus ostreatus* PC15 OX = 1137138 GN = PLEOSDRAFT_1047336 PE = 3 SV = 1	1.457164	0.032554
A0A067NDV5	Carboxylic ester hydrolase OS = *Pleurotus ostreatus* PC15 OX = 1137138 GN = PLEOSDRAFT_46151 PE = 3 SV = 1	1.775427	0.014483
A0A067NFA3	Carboxylic ester hydrolase OS = *Pleurotus ostreatus* PC15 OX = 1137138 GN = PLEOSDRAFT_1051283 PE = 3 SV = 1	1.893255	0.039742
A0A067NL60	Carboxylic ester hydrolase OS = *Pleurotus ostreatus* PC15 OX = 1137138 GN = PLEOSDRAFT_1078816 PE = 3 SV = 1	3.875206	0.002788
A0A067NLN4	Carboxylic ester hydrolase OS = *Pleurotus ostreatus* PC15 OX = 1137138 GN = PLEOSDRAFT_1047807 PE = 3 SV = 1	1.67987	0.000325
A0A067NQW6	Carboxylic ester hydrolase OS = *Pleurotus ostreatus* PC15 OX = 1137138 GN = PLEOSDRAFT_160636 PE = 3 SV = 1	2.516542	0.005202
A0A067NZ51	Carboxylic ester hydrolase OS = *Pleurotus ostreatus* PC15 OX = 1137138 GN = PLEOSDRAFT_1040351 PE = 3 SV = 1	2.404623	5.04 × 10^−7^
A0A067P113	Carboxylic ester hydrolase OS = *Pleurotus ostreatus* PC15 OX = 1137138 GN = PLEOSDRAFT_1091241 PE = 3 SV = 1	2.192162	0.000768
A0A067NHY5	Catalase OS = *Pleurotus ostreatus* PC15 OX = 1137138 GN = PLEOSDRAFT_1090819 PE = 3 SV = 1	0.337985	1.12 × 10^−5^

## Supplementary Materials

The following are available online. Figure S1: Overview of the proteomic result, Table S1: All identified proteins and DEPs, Table S2: GO enrichment of the up-regulated DEPs, Table S3: GO enrichment of the down-regulated DEPs.
